# Magnetic solid phase extraction of Sunitinib malate in urine samples assisted with mixed hemimicelle and spectrophotometric detection

**DOI:** 10.1038/s41598-023-30404-6

**Published:** 2023-02-27

**Authors:** Eslam Pourbasheer, Leila Malekpour, Zhila Azari, Vijay H. Masand, Mohammad Reza Ganjali

**Affiliations:** 1 Department of Chemistry, Faculty of Science, University of Mohaghegh Ardabili, P.O. Box 179, Ardabil, Iran; 2 Department of Chemistry, Payame Noor University, P.O. Box 19395-4697, Tehran, Iran; 3 Department of Chemistry, Faculty of Sciences, Azarbaijan Shahid Madani University, 35 Km Tabriz-Marageh Road, P.O. Box 53714-161, Tabriz, 5375171379 Iran; 4 Department of Chemistry, Vidyabharati Mahavidyalaya, Camp, Amravati, Maharashtra 444602 India; 5 Center of Excellence in Electrochemistry, Faculty of Chemistry, University of Tehran, Tehran, Iran; 6 National Institute of Genetic Engineering and Biotechnology (NIGEB), Tehran, Iran

**Keywords:** Nanoparticles, Medicinal chemistry, Pharmaceutics

## Abstract

The mixed hemimicelle-based solid phase extraction method using the coated sodium dodecyl sulfate by magnetic iron oxide nanoparticles as adsorbent was developed for extraction and determination of Sunitinib malate in real samples prior to determination by UV–Visible spectrophotometry. For the characterization of synthesized nanoparticles, Fourier transform infrared spectroscopy, and scanning electron microscopy was used. The influences of different factors affecting the extraction efficiency of Sunitinib malate, including the pH, the adsorbent amount, the volume and eluent type, the amount of the surfactant, the ionic strength, extraction, and desorption time, were investigated. At the optimized conditions, a good linearity with correlation coefficients of 0.998 and 0.999 was obtained over the concentration ranges of 1–22 and 1–19 µg/mL for water and urine samples, in order. The good recoveries of 97% and 99% and also, the limits of detection equal with 0.9, and 0.8 µg/mL for water and urine samples were enhanced, respectively. These results demonstrate that mixed hemimicelle solid phase extraction is a fast, efficient, economical and selective sample preparation method for the extraction and determination of Sunitinib malate in different water and urine sample solutions.

## Introduction

Sunitinib malate is a novel oral multi-targeted tyrosine kinase inhibitor with established efficacy in treating metastatic renal cell carcinoma and imatinib-resistant gastrointestinal stromal tumor^1–4^. Sunitinib malate prevents the growth of cancer cells by obstructing a group of closely linked tyrosine kinase receptors, containing vascular endothelial growth factor receptors platelet-derived growth factor receptor, FMS-related tyrosine-kinase 3 and stem cell factor receptor (FCF c-KIT) at nanomolar concentrations^5–9^. In addition, SM is approved for the therapy of kidney cancer, advanced renal cell carcinoma, and gastrointestinal stromal tumors^10–12^. Therefore, the determination of Sunitinib malate in water and also in biological fluids has found significance. To date, various analytical techniques have been introduced for the determination of Sunitinib malate, such as LC–MS/MS, HPLC, and high-performance liquid chromatography-UV methods^13–15^. The need for the complicated instruments, on the one hand, and being inexpensive and time-consuming, on the other hand, arouse us to utilize spectrophotometry measurements which presents not only quick analysis but also ease of operation and being economic^16–18^.

An accurate and sensitive procedure such as sample purification and preconcentration are essential prior to instrumental analysis^19–27^. Several pre-treatment methods have been employed for the extraction of Sunitinib malate, including LLE, SPE, and CPE^28–31^. Solid phase extraction has gained more research interest due to being simple, and also flexible in terms of selecting the desirable solid phase as adsorbent, which results in the maximum preconcentration factor, short extraction time and low cost according to the low usage of the organic solvents^32,33^.

More recently, MIONPs have been widely applied in analytical research as a flexible adsorbent for the extraction and determination of various chemical analytes^34–39^. The magnetic nanoparticles provide some advantages over the traditional sorbents, for instance, the high surface area, short diffusion route, high number of surface-active sites, and superparamagnetic features, which lead to the maximum extraction recovery, rapid extraction, and simplicity of extraction for samples with large volumes^40–43^. Nanoparticles are inconstant because of the high level of surface energy, which might end in the nanoparticles agglomeration and less yield of operation^44,45^. To inhibit this problem and improve the extraction efficiency, the surface of the nanoparticles is modified with different organic and, or inorganic materials^46,47^. Surfactants are such organic subcategory that is utilized in this research according to some advantages. First and foremost, SPE is based on the adsorption of surfactant on the surface of the adsorbent, which makes mixed (hemimicelles and, or admicelles) (MHSPE), act as a solid–liquid interface and could be utilized for the extraction and preconcentration of any types of samples from various matrices^17,30,31,48^. Secondly, the modification procedure is simple and could be performed during the extraction; accordingly, it is time-consuming^48^. In this called method, as MHSPE, the ionic surfactants such as SDS, CTAB and, etc., were adsorbed on the surface of the mineral oxides such as, silica, titanium dioxide, alumina, and iron oxides^32,45^. The combination of mixed hemimicelles with solid phase extraction and magnetic nanoparticles has plenty of advantages, such as high extraction yield, regeneration of sorbent, high breakthrough volume, easy analytes elution, and no clean-up steps^31^. Facile process, being fast and modifying at the same time of extraction, another motivational reason to select it as the pretreatment method for most drugs and pollutants^10,33,40,47^.

In this study, modified Fe_3_O_4_ nanoparticles with sodium dodecyl sulfate was formed mixed based solid-phase extraction method, which was successfully employed for extraction and determination of Sunitinib malate in urine and water samples by UV–Visible spectrophotometry technique. According to our literature survey, it is for the first time that the mixed hemimicelles solid phase extraction has been applied for the extraction and determination of Sunitinib malate in urine and water samples.

## Results and discussion

### Characterization of magnetic iron oxide nanoparticles

**Figure 1:**
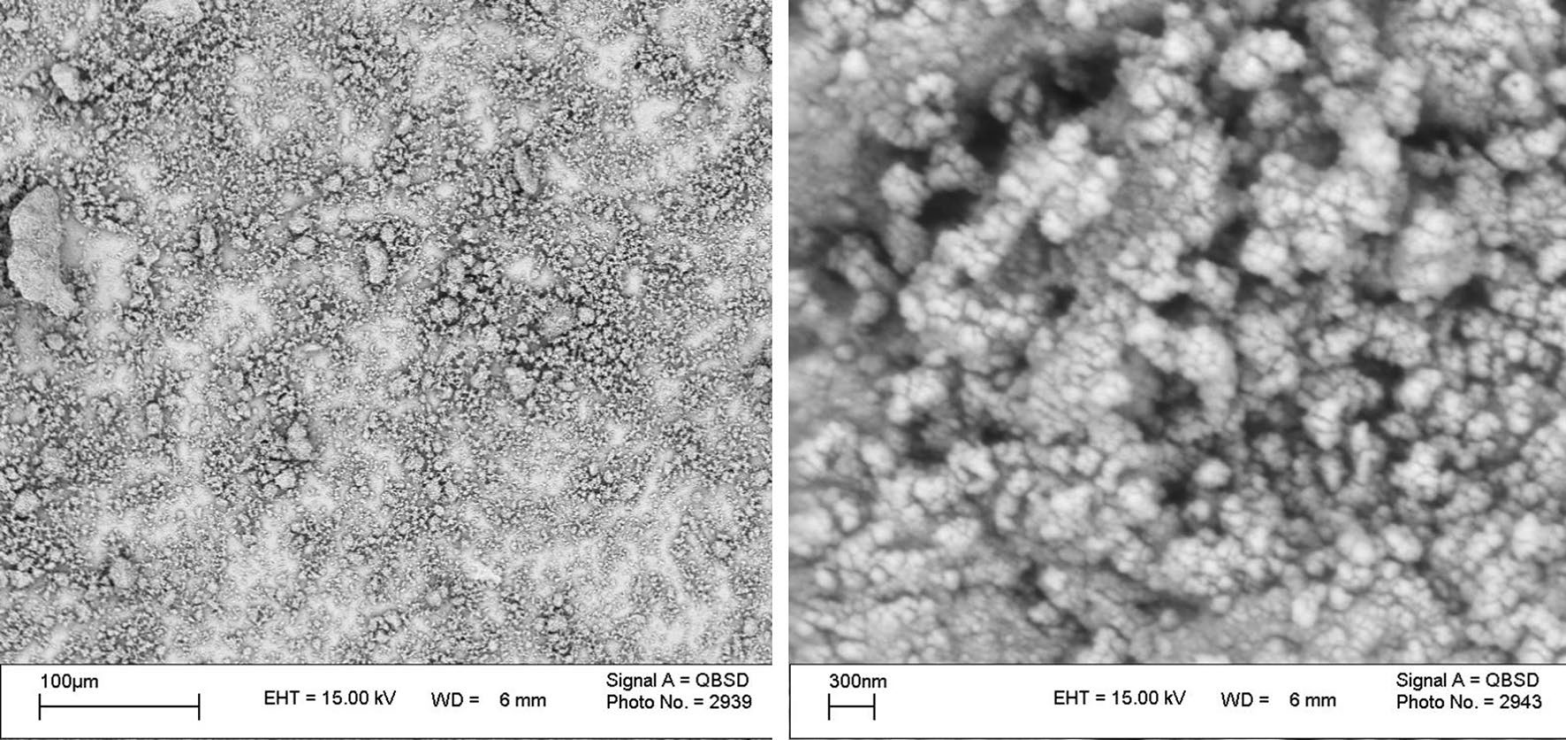
SEM images of SDS-MIONPs nanoparticles.

The SEM and IR were utilized to characterize the synthesized adsorbent, which modified with SDS. According to our previous work^45^, absorption bands appeared at 529, 477 and 3202 cm^-1^ confirm the true synthesis of Fe_3_O_4_ since they are related to the FeO, Fe_2_O_3,_ and the surface OH group of the magnetic nanoparticles, respectively. After modification, new peaks, which stand for the S = O group and stretching mode of the aliphatic C-H groups of SDS were, emerged at 1252 and both 2928 & 2842 cm^−1^, respectively. These peaks demonstrated that the surface of MIONPs was successfully modified with SDS. Furthermore, SEM images (Fig. 1) show the uniform spherical shape of the modified magnetic nanoparticle with the size of about 39–59 nm.

### Mixed hemimicelles-based solid-phase extraction optimization conditions

**Figure 2:**
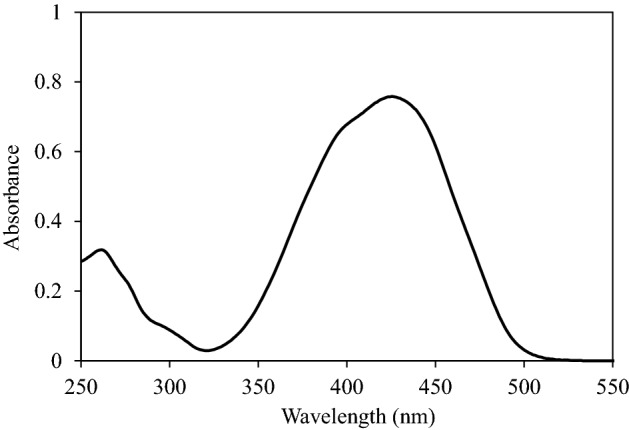
Absorbance spectra of Sunitinib Malate (10 µg/mL).

To maximize the extraction efficiency of Sunitinib malate, the influences of different factors such as the pH, the amounts of adsorbent, and surfactant, the ionic strength, eluent type and volume and extraction /desorption time were investigated and optimized. All the experiments were done three times and the averages of the results were applied for optimization. The UV–vis spectra of Sunitinib malate is shown in Fig. 2. All detections were performed at λ_max_ = 425 nm.

### Effect of pH

**Figure 3:**
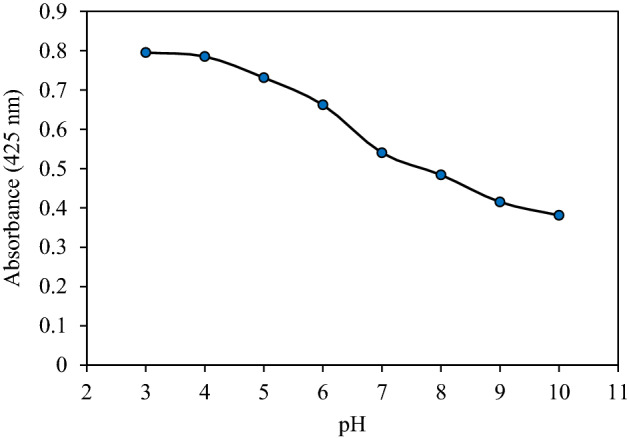
Effect of the pH on extraction efficiency (Extraction conditions: sorbent amount, 30 mg; eluent, 5 mL of methanol; Sample volume: 10 mL; Sample concentration: 10 µg/mL; extraction time, 5 min; desorption time, 5 min).

The most significant parameter affecting the formation of mixed-hemimicells based on the solid adsorbent is the pH of the sample solution^49^. The pH range of 3–10, which was adjusted by applying of 0.1 M HCl or NaOH solution, was investigated. As it is shown in Fig. 3, the highest absorption amount is related to pH 3, and a more increase in the pH from 3 to 10, leads to a reduction in the adsorption of SM. This can be related to the decrease in positive charge density on the MIONPs surface, on the other hand, the positively charged surface of MIONPs in acidic solutions was desirable for the adsorption of anionic surfactants like SDS. The point of zero charge of MIONPs is about 6.5^50^. When the pH of the solution is above the isoelectric point of the MIONPs, the surface charge density of the MIONPs becomes less positive; hence, adsorption of SDS on nanoparticle surfaces is reduced due to the lack of the formation of mixed. Therefore, electrostatic attraction, between the negative head group of SDS as anionic surfactant and the positive surface of MIONPs is potent enough to produce hemimicelles. Despite the mentioned reason, the acidic solution sustains the positive charge of Sunitinib malate and provides the suitable electrostatic attraction with the negative head group of SDS, which is placed at the second layer of mixed hemimicelle. Accordingly, for the optimum value, pH = 3 was selected.

### Effect of SDS amount

**Figure 4:**
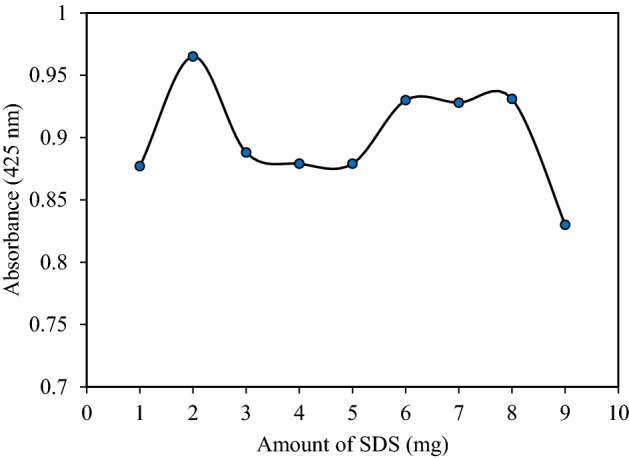
Effect of SDS concentration on extraction efficiency (Extraction conditions: sample pH, 3.0; sorbent amount, 30 mg; eluent, 5 mL of methanol; Sample volume: 10 mL; Sample concentration: 10 µg/mL; extraction time, 5 min; desorption time, 5 min).

The SDS concentration effect, on the extraction efficiency of Sunitinib malate, was considered by adding different concentrations of SDS from 1 to 9 mg/mL to the sample solution. As shown in Fig. 4, in the absence of SDS, the Sunitinib malate hardly adsorbed on the surface of the adsorbent; on the other hand, the adsorption of SM on the surface of magnetic iron oxide nanoparticles is in strict need of modification. The notably increased adsorption is enhanced by the addition of 2 mg of surfactant, which is large enough for the formation of admicelles. By the movements toward smaller amounts of SDS the structure of admicelles changes to mixed hemimicelles and hemimicelles which are not proper for the adsorption of SM. Higher amounts of 2 mg are the beginning point of micelle formation which lead to the gradual decrease in adsorption of SM due to the redistribution of Sunitinib malate in the bulk solution. Therefore, 2 mg of SDS was chosen as the optimum value for the next experiments.

### Effect of ionic strength

The ionic strength of the studied solution on extraction efficiency was evaluated by the utilization of NaCl with various concentrations of 1–10% (w /v). The results indicate that in the presence of salt, especially in extensive amounts, desorption of Sunitinib malate is diminished based on the competitive behaviour of SM and salt for the adsorption on the surface of adsorbent. Accordingly, no salt addition was utilized for the rest of experiments.

### Effect of the MIONPs amount

**Figure 5:**
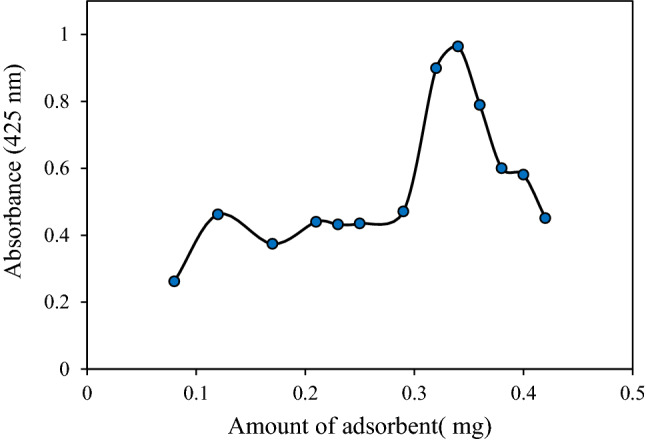
Effect of MIONPs amount on extraction efficiency (Extraction conditions: sample pH, 3.0; SDS concentration:2 mg/mL; eluent, 5 mL of methanol; Sample volume: 10 mL; Sample concentration: 10 µg/mL; extraction time, 5 min; desorption time, 5 min).

The amount of the adsorbent definitely affects the mechanism of adsorption and extraction efficiency. In order to choose the required quantity of adsorbent (MIONPs) for the extraction of Sunitinib malate, different concentrations of MIONPs suspension were used in the range of 0.08–0.42 mg/mL. Nanoparticles have a higher surface area than ordinary sorbents (micron-size particle sorbents), which result in high extraction capacity and fast extraction dynamics. Therefore, satisfactory results can be obtained with fewer amounts of sorbents. As shown in Fig. 5, the extraction recovery of Sunitinib malate increased with increasing sorbent amounts from 80 up to 320 mg/mL of Fe_3_O_4_ nanoparticles, due to the increased accessible adsorption sites. The more addition of the adsorbent diminished the adsorption of SM, due to the dramatic reversal in admicelle formation, which ended in mixed and, or hemimicelles structures. Therefore, 320 mg/mL of the magnetic adsorbent amount was selected as the optimum.

### The influence of kind and the volume of the eluent

**Figure 6:**
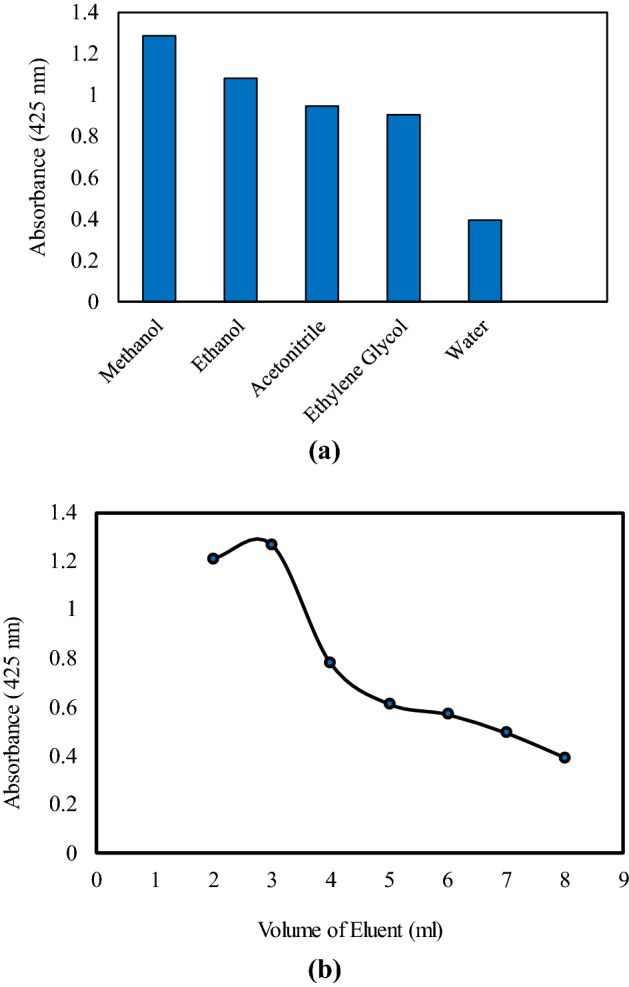
Effect of eluent type (**a**) and eluent volume (**b**) on extraction efficiency (Extraction conditions: sample pH, 3.0; sorbent amount, 320 mg; SDS concentration:2 mg/mL; eluent volume, 5 mL; Sample volume: 10 mL; Sample concentration: 10 µg/mL; extraction time, 1 min; desorption time, 7 min).

To enhance the maximum release of SM, different organic solvents such as acetonitrile, ethanol, methanol, ethylene glycol and water were studied. As shown in Fig. 6a, the highest extraction recovery of Sunitinib malate was obtained by the use of methanol which conforms to the necessity of the utilization of organic solvent for the disruption of admicelles, structure. Furthermore, the highest solubility of SM in methanol in comparison with others might be the reason for the highest absorbance. Figure 6b exhibits the effect of different volumes of methanol in the range from 2 to 8 mL on the extraction efficiency of Sunitinib malate. By incrementing the volume to 3 mL, the extraction efficiency increased, and the diminution was observed for the higher amounts 3 to 8 ml. It would attribute to the analytes dilution in the high volumes of the eluent. Hence, for the optimum eluent volume, 3 mL of methanol was selected. Moreover, our primary solution, which extracted was acidic, and therefore, 3 ml of the basic solution (methanol: water; 94:6 v/v) demonstrated the best extraction efficiency, and was selected as the desirable extractor.

### The impact of time on both extraction and back extraction

Extraction/back extraction time is defined as the needed time to bring both solution and adsorbent into contact in order to enhance the complete transfer of analyte to adsorbent/extractor solvent and in solid phase extraction is measured as the time for the shaking by the application of a shaker^45^. This extraction/ back extraction time must be long enough to make solution/adsorbent approximately bare of analyte. In this study, the effect of extraction and back extraction times on the extraction efficiency of Sunitinib malate were investigated in the range of 1–5 and 1–9 min, respectively. The results showed that the highest extraction efficiency was obtained in the shortest tested time as extraction time (1 min). After 1 min, the adsorption remained constant with less reduction. Also, the maximum desorption was observed at 7 min. The desorption's were incomplete in the times shorter than 7 min and then decreased. The rapid dynamic process and high surface area of MIONPs along with homogeneous distribution of the nano-sorbent throughout the sample and could be the possible reasons for gaining a rapid extraction process. In a word, MIONPs-based MHSPE is a method with the capability of a fast separation procedure which leads to a short analysis time. Therefore, 1 and 7 min were selected as the optimized adsorption and desorption time.

### Analytical performance

In order to validate the proposed method, at the optimized experimental conditions, some figures of merits such as the limit of detection and quantification, the linear range of calibration curve, correlation of determinations (R^2^), and the accuracy and precision of the method were examined. The analytical performances of the presented method are presented in Table 1. The calibration curve was linear in the range from 1 to 22 and 1 to 19 µg/mL with determination coefficients of 0.998 and 0.0999 for water and urine samples, respectively. The LOD and LOQ were defined according to the IUPAC recommendation as follows: LOD = 3.3 (SD/S) and LOQ = 10 (SD/S) where SD is the standard deviation of the blank and S is the slope of calibration curve. The LODs were 0.9 and 0.8 µg/mL and also the LOQs were 2.9 and 2.6 µg/mL for determination of Sunitinib malate in water and urine samples, respectively. Moreover, to determine the relative standard deviation (%) of the analytical method five replicates were done for each concentration. The method accuracy was investigated by the measurement of relative error which is 100 multiplied by the resulting subtraction of founded and added concentration of SM in spiked samples divided to added concentrations. As the results reveal in Table 1, the proposed method exhibited good linearity, appropriate precision and low LOD and LOQ for the determination of the analyte.

**Table 1:** Figures of merit for the applied MHSPE in real samples.

Sample	LOD (µg/mL)	LOQ (µg/mL)	Regression equation	R^2^	EP (%)^a^	LDR (mg/L)^b^
Water	0.9	2.9	y = 0.0362x + 0.1134	0.998	97	1–22
Urine	0.8	2.6	y = 0.0415x + 0.0031	0.999	99	1–19

^a^Extraction percentage.

^b^Linear dynamic range.

### Real sample analysis

Under the optimized conditions, the mixed hemimicelles SPE method was applied for the extraction and determination of Sunitinib malate in spiked tap water and urine samples. Since Sunitinib malate was not detected in the real samples, 10 µg/mL of Sunitinib malate was added into the water and urine samples, and extraction was done based on the procedure. The analytical results are summarized in Table 2. It can be seen, that the enhanced recoveries are about 97% and 99% for water samples and urine samples, respectively, which indicate the suitability of MIONPs sorbents for selective extraction and determination of Sunitinib malate in real samples. Also, the relative standard deviations (RSDs %) for five replicate extractions were 0.67 and 0.41% and the gained relative errors were 3.1 and 0.04 for water and urine, respectively; indicating the proper precision and accuracy of the method. Also, the performance of the proposed method was compared to that of other reported methods for determination of Sunitinib Malate and the results are presented in Table 3.

**Table 2:** Determination of Sunitinib Malate (SM) in real samples.

Sample	C_added_ (µg/mL)	C_found_ (µg/mL)	RSD (%) (n = 5)	R^2^	Relative recovery (%)	Relative error (%)
Water	10	10.31	0.67	0.998	97	+ 3.1
Urine	10	10.60	0.41	0.999	99	+ 6.04

**Table 3:** Comparison of the proposed MHSPE method with some of the methods reported in the literature for determination of Sunitinib Malate.

Sample	Detection system	LOD (ng/mL)	LOQ (ng/mL)	LDR (ng/mL)^b^	R^2^	References
Plasma	LC–MS/MS	–^a^	1.37	1.37–1000	0.99	^4^
Plasma	LC–MS/MS	–	2	2—250	0.9919	^10^
Plasma	LC–MS/MS	–	0.10	0.10—150	0.998	^12^
Plasma	HPLC–UV–Vis	–	10	20–200	–	^13^
Plasma	LC–MS/MS	–	0.2	0.2–500	0.9950	^15^
Urine	UV–Visible	0.8 (mg/L)	2.6 (mg/L)	1–19 (mg/L)	0.999	This work
Water	UV–Visible	0.9 (mg/L)	2.9 (mg/L)	1–22 (mg/L)	0.998	This work

^a^Data not reported.

^b^Linear dynamic range.

## Conclusion

The proposed mixed hemimicelles solid phase extraction was developed for extraction and determination of Sunitinib malate in tap water and urine samples prior to spectrophotometric determination. Sodium dodecyl sulfate was utilized for the modification of magnetic iron oxide nanoparticles and the construction of mixed hemimicelles. The proposed method provides various advantages like short analysis time, simplicity, low cost, high extraction efficiency, low detection limits and proper recoveries. This method presents satisfactory repeatability and accuracy for the extended dynamic linear ranges of 1–22 and 1–19 µg/ml for water and urine samples, respectively. Also, the satisfactory recoveries and precision of the proposed MHSPE method indicate that MIONPs sorbents have considerable potential for the extraction and determination of Sunitinib malate from biological fluids. Finally, this method was handled for designation of SM in water and urine samples with low detection limits and proper recoveries of 97 and 99, respectively. Hence, the proposed analysis method could be successfully applied for extraction and determination of drugs in real samples.

## Experimental

### Materials and instruments

**Figure 7:**
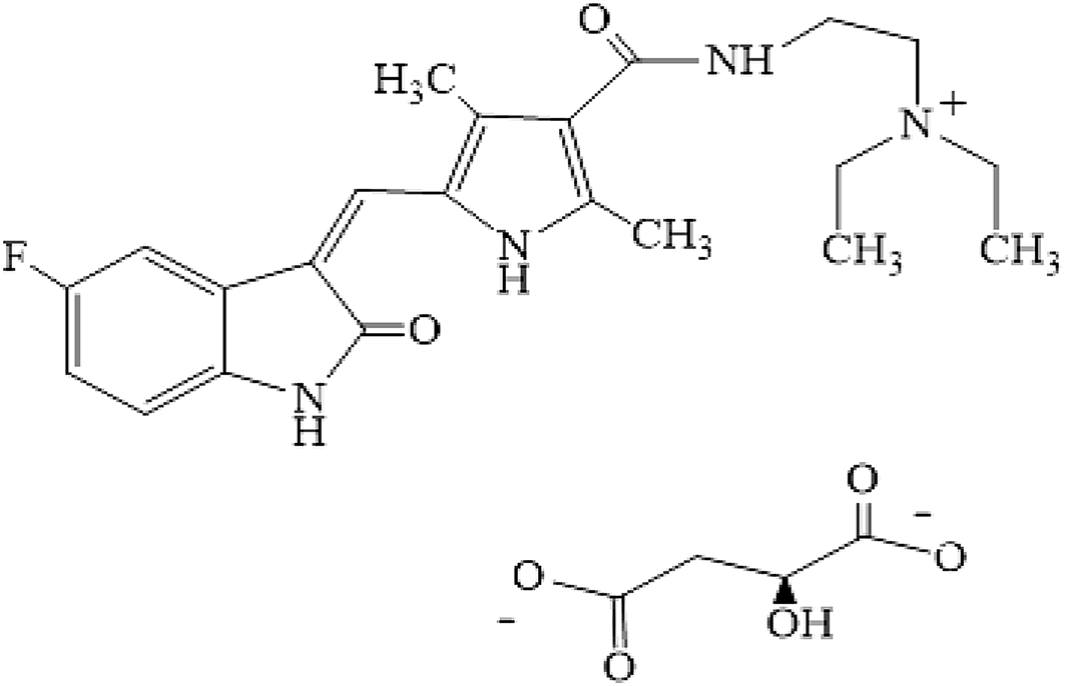
Structure of Sunitinib Malate (SM).

All used reagents were of analytical grade and distilled water was used for making of all the aqueous solutions. The FeCl_3_.6H_2_O, FeCl_2_.4H_2_O, SDS, NaOH (99%), HCl (37%), aqueous ammonia (25 wt%), NaCl, methanol, ethanol, ethylene glycol, acetonitrile, dimethyl sulfoxide and Sunitinib malate were purchased from Merck (Darmstadt, Germany). The molecular structure of Sunitinib malate is presented in Fig. 7. A stock solution of the Sunitinib malate (100 mg/L) was prepared by dissolving proper amount of Sunitinib malate in dimethyl sulfoxide and methanol and stored at 4 °C. All the solutions were prepared by suitable diluting of the stock solution with distilled water. The Climo-Shaker ISF1-X (Kuhner AG, Switzerland) was used for shacking of the mixtures. A Jenway model 4510 (Stone, UK), pH meter with a glass electrode was applied for the pH measurements. All of spectrophotometric measurements of the solutions were done by a Analytic Jena SPECORD 250 UV–vis spectrophotometer (Germany). The UV detection of Sunitinib malate was performed at 425 nm. The FT-IR spectra of the prepared nanoparticles was carried out by a Shimadzu prestige-21 (Japan) FT-IR spectrophotometer in the range of 400–4000 cm^-1^. The structure of the synthesized nanoparticles was characterized by a scanning electron microscopy (SEM, LEO 1430VP).

### Magnetic iron oxide nanoparticles preparation

The nanoparticles of Fe_3_O_4_ were synthesized with the help of a conventional co-precipitation method similar to our previous works^45^. The 4.3 g of FeCl_2_·4H_2_O and 11.68 g of FeCl_3_.6H_2_O were dissolved in 200 mL of deionized water under a nitrogen atmosphere at 85 °C. An ammonia solution (20 mL, 30% w/w) was added dropwise into the mixture with vigorous stirring (400 rpm) for 30 min. The orange color of the solution changed to black quickly. After cooling down to room temperature, the obtained MNPs precipitate was separated from the reaction mixture under the magnetic field. The magnetite precipitates were eluted twice with deionized water, and once with 0.02 mol/L sodium chloride.

### Preparation of real samples

The efficiency of this method was evaluated by the application of water and urine as real samples. Tap water samples were freshly prepared from our analytical laboratory (Ardabil, Iran). Water samples were just filtered with a Millipore filter before extraction. The urine samples were taken from a voluntary female. The collected urine samples were centrifuged at 2500 rpm for 20 min at room temperature to sediment white lipidic solid, then filtered with a 0.2 µm syringe filter. The spiked urine samples were diluted 1:10 using distilled water, in order to decrease the matrix effect.

### Ethics declaration

The urine sample preparation method was carried out in accordance with relevant guidelines and regulations. The urine sample was taken from a voluntary female and informed consent of participating subject, was obtained. The researchers institute did not require approval for the use of urine in experiments in this instance.

### Magnetic MHSPE procedure

**Figure 8:**
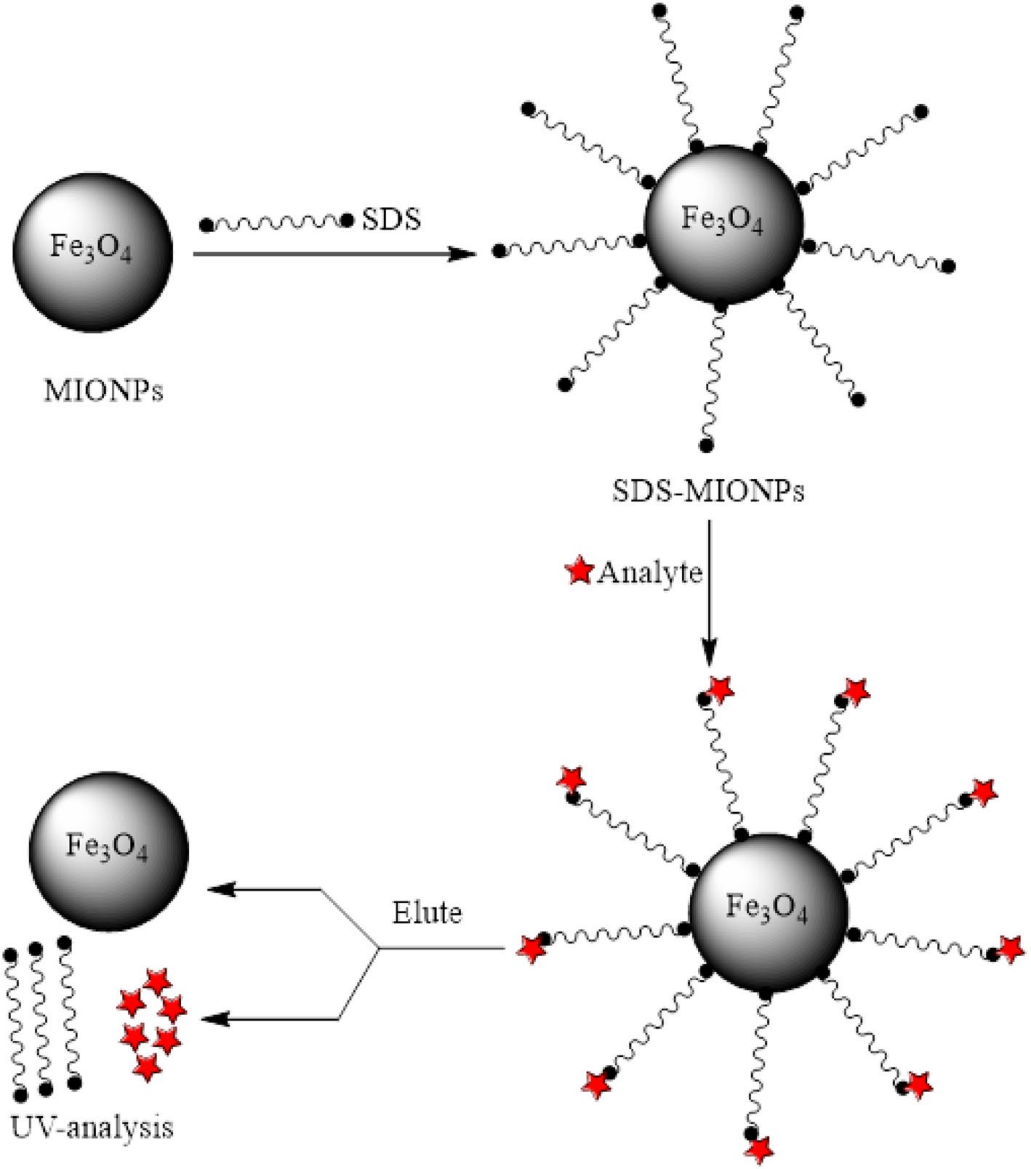
A schematic diagram of magnetic MHSPE procedure.

The step-by-step procedure to accomplish MHSPE of Sunitinib malate, was as follows (Fig. 8): a 10 mL of the aqueous solution containing Sunitinib malate (10 µg/mL) was transferred into a 25 mL beaker. Next, an amount of 0.5 mL (30 mg/mL) of Fe_3_O_4_ NPs suspension and 0.2 mL of the SDS solution (2 mg/mL) were sequentially added. In order to complete the extraction process, the mixtures were shacked and permitted for 5 min. After that, the SDS-coated MIONPs were isolated from the solutions using an external magnet. After 2 min, the solutions became clear and supernatant solutions were decanted. The 5 mL of methanol was used for the elution of preconcentrated analyte from the isolated adsorbent. The concentration of Sunitinib malate was determined at a wavelength of 425 nm using a UV–Vis spectrophotometer.

## Data Availability

All data supporting the conclusions of this research article are included within the manuscript.

## Abbreviations

MHSPE: Mixed hemimicelle-based solid phase extraction
SDS: Sodium dodecyl sulfate
SDS-MIONPs: Sodium dodecyl sulfate—magnetic iron oxide nanoparticles
SM: Sunitinib malate
FT-IR: Fourier transform infrared spectroscopy
SEM: Scanning electron microscopy
LOD: Limits of detection
LOQ: Limit of quantification
TKI: Tyrosine kinase inhibitor
VEGFR: Vascular endothelial growth factor receptors
PDGF: Platelet derived-growth factor receptor
GIST: Gastrointestinal stromal tumors
LC–MS/MS: Liquid chromatography-tandem mass spectrometry
HPLC: High-performance liquid chromatography
LLE: Liquid–liquid extraction
SPE: Solid-phase extraction
CPE: Cloud point extraction
MIONPs: Magnetic iron oxide nanoparticles
CTAB: Cetyltrimethyl ammonium bromide
PZC: Point of zero charge
FeCl_3_·6H_2_O: Ferric chloride hexahydrate
FeCl_2_·4H_2_O: Ferrous chloride tetrahydrate
NaOH: Sodium hydroxide
HCl: Hydrochloric acid
NaCl: Sodium chloride
